# Increased expression of IL-1β in adipose tissue in obesity influences the development of colon cancer by promoting inflammation

**DOI:** 10.1007/s13105-024-01048-5

**Published:** 2024-09-21

**Authors:** Gabriela Neira, Javier Gómez-Ambrosi, Javier A. Cienfuegos, Beatriz Ramírez, Sara Becerril, Amaia Rodríguez, María A. Burrell, Jorge Baixauli, Amaia Mentxaka, Marcos Casado, Camilo Silva, Javier Escalada, Gema Frühbeck, Victoria Catalán

**Affiliations:** 1 Metabolic Research Laboratory, Clínica Universidad de Navarra, Avda. Pío XII, 36, 31008 Pamplona, Spain; 2 Present Address: CIBER Fisiopatología de la Obesidad y Nutrición (CIBEROBN), Instituto de Salud Carlos III, Pamplona, Spain; 3 Obesity and Adipobiology Group, Instituto de Investigación Sanitaria de Navarra (IdiSNA), Pamplona, Spain; 4 Department of Surgery, Clínica Universidad de Navarra, Pamplona, Spain; 5 Department of Histology & Pathology, Universidad de Navarra, Pamplona, Spain; 6 Department of Endocrinology & Nutrition, Clínica Universidad de Navarra, Avda. Pío XII, 36, 31008 Pamplona, Spain

**Keywords:** IL-1β, Obesity, Colon cancer, Inflammation, Intestinal permeability

## Abstract

Excess adiposity contributes to the development of colon carcinoma (CC). Interleukin (IL)-1β is a pro-inflammatory cytokine relevant in obesity-associated chronic inflammation and tumorigenic processes. We herein aimed to study how obesity and CC affects the expression of *IL1B*, and to determine the impact of IL-1β on the regulation of metabolic inflammation and gut barrier function in the context of obesity and CC. Samples from 71 volunteers were used in a case–control study and a rat model of diet-induced obesity (DIO). Furthermore, bariatric surgery was used to determine the effect of weight loss on the intestinal gene expression levels of *Il1b*. To evaluate the effect of IL-1β and obesity in CC, we treated the adenocarcinoma cell line HT-29 with IL-1β and the adipocyte-conditioned medium (ACM) from patients with obesity. We showed that obesity (*P* < 0.05) and CC (*P* < 0.01) upregulated the transcript levels of *IL1B* in visceral adipose tissue as well as in the colon from patients with CC (*P* < 0.01). The increased expression of *Il1b* in the ileum and colon in DIO rats decreased after weight loss achieved by either sleeve gastrectomy or caloric restriction (both *P* < 0.05). ACM treatment on HT-29 cells upregulated (*P* < 0.05) the transcripts of *IL1B* and *CCL2*, while reducing (*P* < 0.05) the expression of the anti-inflammatory *ADIPOQ* and *MUC2* genes. Additionally, IL-1β upregulated (*P* < 0.01) the expression of *CCL2* and *TNF* whilst downregulating (*P* < 0.01) the transcript levels of *IL4*, *ADIPOQ* and *TJP1* in HT-29 cells. We provide evidence of the important role of IL-1β in obesity-associated CC by directly promoting inflammation.

**Supplementary Information:**

The online version contains supplementary material available at 10.1007/s13105-024-01048-5.

## Introduction

Obesity is considered a public health concern at all ages [15], which has led attention to its different comorbidities with an emphasis on type 2 diabetes (T2D), cardiovascular diseases as well as different types of cancers [6, 67]. Specifically, obesity has been established as a risk factor for the development of colon carcinoma (CC) [37, 38]. Consequently, the increased prevalence of obesity has been linked to an earlier development of CC among young adults, and has contributed to make CC the third most common cancer worldwide [47, 60]. Despite a great amount of evidence having established a link between excess adiposity and CC development [62], cellular and molecular mechanisms describing this association are limited. Different systemic alterations linked to obesity, including hormonal disturbances, insulin resistance, dyslipidemia or hyperglycemia induce carcinogenesis, but other factors, such as chronic inflammation, dysbiosis and altered intestinal barrier, have been recently considered of crucial relevance to CC development [39, 65].

Chronic inflammation attributed to dysfunctional adipose tissue (AT) in obesity [31] is fundamental in the pathogenesis of CC, promoting a preneoplastic microenvironment that favors tumor development and invasion [45, 65]. Damaged AT in obesity leads to hypoxia, cell death, immune cell infiltration, and the activation of different inflammatory pathways together with their corresponding pro-inflammatory mediators [57]. With this process, damage-associated molecular pattern molecules (DAMPs) are released from the adipocytes, activating toll-like receptors and inflammasomes and thus, perpetuating a pro-inflammatory response mediated by different cytokines, including interleukin (IL)-1β [23, 64]. IL-1β forms part of the IL-1 superfamily, a classic inflammatory and pyrogenic group of interleukins [44]. IL-1β is mainly released by myeloid cells including monocytes and macrophages, dendritic cells, granulocytes and myeloid-derived suppressor cells, in response to endogenous and exogenous danger signals [68]. Although the activation of IL-1β is necessary to resolve acute inflammation processes, the sustained release of IL-1β in obesity maintains both a constant activation of pro-inflammatory cascades [43] as well as an enhanced expression of pro-tumorigenic mediators including nitric oxide (NO), cyclooxygenase-2 (COX-2), chemokines and metalloproteinases (MMPs) [53] favoring tumor development [12]. In addition, circulating levels of IL-1β have been found to be increased in patients with CC as well as with other types of cancers including gastroesophageal cancer, squamous cell carcinoma or breast cancer [54]. In this line, IL-1β increases tumor progression in murine models and is considered a mediator of chronic non-resolving inflammation linked to gastrointestinal tract malignancies [44].

Since obesity has been established as a major risk factor for CC development, strategies promoting weight loss such as caloric restriction (CR) and bariatric surgery (BS) may improve the prognosis of patients with obesity and cancer [58]. Reportedly, BS compared to non-surgical approaches has been shown to decrease the incidence of different obesity-associated cancers including CC [2, 51]. These findings have been corroborated by the Swedish Obese Subjects Study (SOS), which reported an association between BS and a reduction in cancer risk [59]. Importantly, BS also resulted in an improved insulin response and a decrease in systemic inflammation. A potential mechanism underlying the reduced cancer risk and the metabolic benefits associated with BS, may be attributed to molecular changes that affect the inflammatory burden and the gene expression levels in the AT from patients after surgery [13, 28]. Serum concentration levels of different inflammatory markers, such as C-reactive protein (CRP) [14], IL-1 receptor antagonist [18], or IL-36 [24] decreased after BS, reducing inflammation and improving the metabolic profile of the individuals.

Obesity-associated chronic inflammation is a well-known risk factor for CC development. Nonetheless, the direct mechanisms that link dysfunctional AT with CC remain undetermined. For this reason, we hypothesized that obesity and CC may influence the expression of IL-1β and its related molecules in the colon and visceral AT (VAT) from patients with obesity and CC. The effect of weight loss on the gene expression levels of *Il1b* was studied in the intestinal tract of a rat model with diet-induced obesity (DIO). To determine the direct effect that IL-1β may have on the inflammatory response in cancer, the colon adenocarcinoma cell line HT-29 was treated with IL-1β. Moreover, to assess the impact of obesity on the expression of *IL1B* in CC cells, we treated the HT-29 cell line with the adipocyte-conditioned medium (ACM) obtained from patients with obesity.

## Material and methods

### Patient samples

VAT samples were obtained from 71 patients [24 normal-weight (NW) (13 with CC) and 47 with obesity (OB) (13 with CC)] that attended the Department of Endocrinology & Nutrition and Surgery at the Clínica Universidad de Navarra. Patients were classified as NW or with OB according to the body mass index (BMI) criteria (weight in kilograms divided by the square of height in meters), considering NW a BMI < 25 kg/m^2^ and OB a BMI ≥ 30 kg/m^2^. Body fat percentage was predicted using the Clínica Universidad de Navarra-Body Adiposity Estimator (CUN-BAE) [29]. Volunteers underwent a clinical assessment prior to the inclusion in the study, including analysis of anamnesis and physical examination. CC patients were classified following the diagnostic protocol for CC and underwent a curative resection surgery at the aforementioned Clinic (Table S1). The written informed consent of all participants was obtained. In an ethical and scientific regard, the experimental design was approved by the Clínica Universidad de Navarra’s Ethics Committee responsible for research (2018.094). A cohort of human colon total RNA samples (7 from normal colon tissue and 11 from CC tissue) were acquired from OriGene (Rockville, MD, USA). The clinical information associated with these samples is available from the OriGene web site.

### Blood processing and assays

Blood samples were collected by venipuncture after an overnight fast and centrifuged at 12,000 *g* for 15 min at 4 ºC to obtain serum and plasma. Glucose and triglycerides as well as carcinoembryonic antigen (CEA) and CRP were determined as previously described [7]. An automated cell counter (Beckman Coulter, Inc., Fullerton, CA, USA) was used to determine the white blood cell (WBC) count. Circulating levels of IL-1β were assessed by a commercially available ELISA kit (RayBiotech, Inc., Norcross, GA, USA) according to the manufacturer’s instructions with intra- and inter-assay coefficients of variation of < 10 and < 12%, respectively.

### Animal model of diet-induced obesity and bariatric surgery

Eight-week-old male Wistar rats (*n* = 38) were fed *ad libitum* with a high-fat diet (HFD) (*n* = 28) (Diet F3282, Bio-Serv, Frenchtown, NJ, USA) or a normal chow diet (ND) (*n* = 10) (Diet 2014S, Harlan Laboratories Inc., Barcelona, Spain) for 16 weeks. Body weight and food intake were weekly registered as previously described [45]. Subsequently, rats in the HFD group were randomized to undergo either sham surgery (*n* = 6), sleeve gastrectomy (SG) (*n* = 6) or pair-fed (PF) (*n* = 6). The PF group ate the corresponding amount of food eaten by the rats in the SG group to differentiate subsequent effects attributed to a decrease in food intake after surgery. After interventions, animals were fed a ND. Anesthesia and surgical procedures were performed as previously described [66]. After all interventions, rats were sacrificed by decapitation and the duodenum, jejunum, ileum and colon were dissected out and stored at -80 ºC. The Ethical Committee for Animal Experimentation of the University of Navarra (026/19) approved all experimental protocols, and these followed the European Guidelines for the Care and Use of Laboratory Animals (directive 2010/63/EU).

### Cell cultures

The colorectal adenocarcinoma cell line HT-29 (HTB-38™) was obtained from the ATCC (Middlesex, UK) and cultured in accordance with the manufacturer’s guidelines in McCoy’s 5A (1x) GlutaMAX modified medium (Gibco, New York, NY, USA) supplemented with 10% new-born calf serum (NCS) (Gibco), antibiotic–antimycotic (Merck, Darmstadt, Germany), and 20 μL of β-mercaptoethanol (Merck). Cell cultures were incubated at 37 ºC and 5% CO_2_, and the medium was changed every 2 days. Cells (1 × 10^6^/well) were seeded into 6-well plates during 24 h and were serum-starved for 2 h prior to the corresponding stimuli. Afterwards, cells were treated with different concentrations of IL-1β (R and D Systems, Minneapolis, MN, United States) (1, 10 and 100 ng/mL) or adipocyte conditioned media (ACM) (20% and 40%) for 24 h.

To obtain the ACM, visceral AT samples were taken from patients with OB. Cells from the stromal vascular fraction (SVFC) were isolated and seeded into 2 × 10^5^ cells/well and cultured in DMEM/F-12 [1:1] medium (Gibco), supplemented with 17.5 mol/L of glucose, 16 mmol/L of biotin, 18 mmol/L of pantothenate, 100 mmol/L of ascorbate, 100 mmol/L of antibiotic antimycotic (Merck) and 10% NCS (Gibco). Medium was changed after 4 days to an adipocyte medium supplemented with 3% NCS, 0.5 mmol/L of 3-isobutyl-1-methylxanthine (IBMX), 0.1 mmol/L of dexamethasone, 1 mmol/L of BRL49653 and 10 mg/mL of insulin, for corresponding adipocyte differentiation induction. After 3 days, cells were grown in the aforementioned medium without IBMX and BRL49653 for a remaining total of 7 days to achieve cell differentiation. ACM was collected from these cultures at the end of the 7th day, centrifuged, diluted (20% and 40%) and stored at -80 ºC for further experiments. In order to assess the concentration of the secreted factor IL-1β in the ACM, a commercially available ELISA kit (RayBiotech, Inc) was used according to the manufacturer’s instructions.

### RNA isolation and real-time PCR

RNA extraction for HT-29 as well as rat tissue samples was performed using TRIzol^**®**^ Reagent (Invitrogen, Carlsbad, CA, USA) and QIAzol^**®**^ (Qiagen, Hilden, Germany) for human VAT samples. A total of 3 μg of RNA was then reverse-transcribed into cDNA, using hexamers as random primers and M-MLV reverse transcriptase (Invitrogen), obtaining a final volume of 60 μL. Transcript levels were quantified using Real-Time PCR (7300 Real Time PCR System, Applied Biosystems, Foster City, CA, USA). The corresponding primers and probes for gene expression analysis were designed using Primer Express 2.0 (Applied Biosystems) software (Table S2). The cDNA was amplified following these conditions: 95 °C for 10 min, 45 cycles of 15 s at 95 °C and 1 min at 59 °C, using the TaqMan^**®**^ Universal PCR Master Mix (Applied Biosystems). Eukaryotic *18S* rRNA (Applied Biosystems) was used as endogenous control to normalize all samples studied. Each sample was run in triplicate and the average values were calculated. The corresponding mRNA expression was shown as a fold increase compared to the *18S* rRNA endogenous expression levels.

### Statistical analysis

Data are presented as mean ± standard error of the mean (SEM). Due to its non-normal distribution, gene expression levels were logarithmically transformed. All statistical analyses were performed using IBM SPSS Statistics v22 software (SPSS Inc, Chicago, IL, USA) and corresponding graphs were made using GraphPad Prism v9 (GraphPad Software, Inc., San Diego, CA, USA). Differences between groups were studied using unpaired Student’s *t*-test, two-way analysis of covariance (ANCOVA) or one-way ANOVA followed by Tukey’s post hoc tests when applicable. Statistical significance was determined by *P* < 0.05.

## Results

### Obesity and CC increased the expression of *IL1B* in colon and VAT

Anthropometric and biochemical data of the patients involved in the study are shown in Table 1. As expected, body weight, BMI and estimated BF were higher in patients with OB (*P* < 0.01). However, all anthropometric values regarding adiposity were lower (*P* < 0.01) in patients with OB and CC compared with patients with OB without CC, suggesting certain degree of cachexia. Circulating concentrations of fasting glucose and triglycerides were increased in both groups of patients with OB. Serum levels of CRP, a classic biochemical marker of inflammation, were higher in patients with OB and CC. Regarding CEA, a characteristic CC tumor marker [40], increased levels were found in patients with CC.

**Table 1:** Anthropometric and biochemical characteristics of patients included in the study

	Normal weight		Obesity				
	non-CC	CC	non-CC	CC	*P* Obesity	*P* Cancer	*P* OB x CC
n (male, female)	11 (6, 5)	13 (7, 6)	34 (13, 21)	13 (7, 6)	-	-	-
Age (years)	45 ± 6	60 ± 2	49 ± 2	64 ± 3	0.238	< 0.001	0.934
Body weight (kg)	59.6 ± 2.8	64.5 ± 2.1	108.4 ± 3.2^**,§§^	80.3 ± 2.9^**,§§,‡‡^	< 0.001	0.003	< 0.001
Body mass index (kg/m^2^)	20.8 ± 0.5	21.8 ± 0.4	38.2 ± 1.1^**,§§^	31.4 ± 0.7^**,§§,‡‡^	< 0.001	< 0.001	< 0.001
Estimated BF %	22.4 ± 2.2	28.2 ± 1.6	44.4 ± 1.4^**,§§^	35.3 ± 1.7^**,§§,‡‡^	< 0.001	0.022	< 0.001
Fasting glucose (mg/dL)	88 ± 5	130 ± 4	125 ± 9	127 ± 8	0.258	0.231	0.086
T2D (%)	0	38	37	55			
Triglycerides (mg/dL)	71 ± 11	94 ± 2	133 ± 11	182 ± 19	0.009	0.369	0.598
C-reactive protein (mg/L)	0.16 ± 0.06	1.02 ± 0.65	0.81 ± 0.22	9.16 ± 0.67^**,§§,‡‡^	< 0.001	< 0.001	< 0.001
CEA (ng/mL)	1.58 ± 0.32	2.55 ± 0.44	1.68 ± 0.28	8.41 ± 2.60	0.267	0.021	0.401
Leucocyte (× 10^9^/L)	6.36 ± 0.51	6.82 ± 1.03	7.02 ± 0.44	7.72 ± 0.81	0.568	0.473	0.541

Data are mean ± SEM

Statistical differences were analyzed by two-way ANCOVA and one-way ANOVA followed Tukey’s *post hoc* tests in case of interaction between factors

*BF* body fat, *CC* colon cancer, *CEA c*arcinoembryonic antigen, *OB* obesity, *NW* normal weight, *T2D* type 2 diabetes

^**^P < 0.01 *vs* NW non-CC, ^§§^*P* < 0.01 *vs* NW CC, ^‡‡^*P* < 0.01 *vs* obesity non-CC

**Fig. 1:**
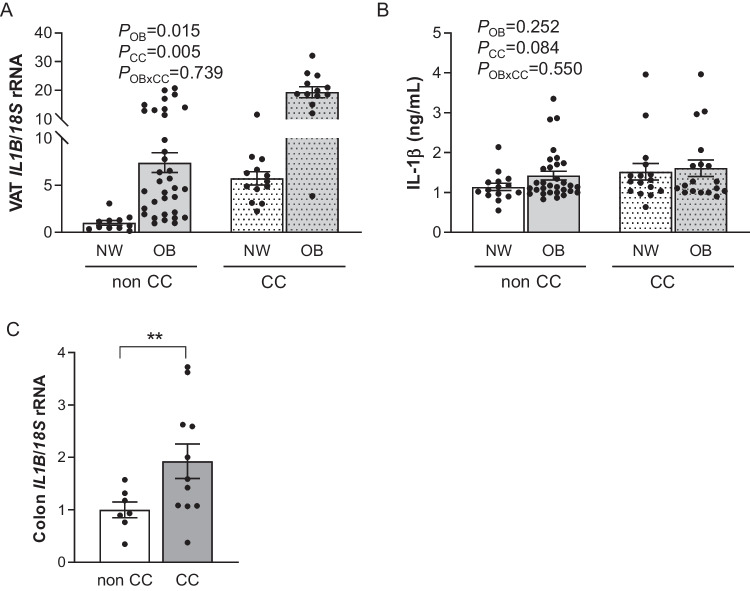
(**A)** Gene expression levels of *IL1B* visceral adipose tissue (VAT) and (**B**) circulating levels of IL-1β from normal weight (NW) volunteers and patients with obesity (OB) classified according to the presence or absence of colon cancer (CC) [NW-nonCC: *n* = 11; OB-non CC: *n* = 34; NW-CC: *n* = 13; OB-CC: *n* = 13]. (**C**) *IL1B* mRNA levels in the colon from patients with and without CC [nonCC: *n* = 7; CC: *n* = 11]. Differences between groups were analyzed by two-way ANCOVA and by two-tailed unpaired Student’s *t*-tests. Bars represent the mean ± SEM. ^**^P < 0.01

We found a significant upregulation in the expression levels of *IL1B* in VAT due to OB (*P* < 0.05) and CC (*P* < 0.01) (Fig. 1A). Although patients with CC exhibited a tendency towards increased circulating levels of IL-1β, differences were not statistically significant (Fig. 1B). Based on these results, we aimed to analyze the expression of *IL1B* in the colon tissue from patients with CC compared to control patients. As shown in Fig. 1C, the gene expression levels of *IL1B* were upregulated in the colon from patients with CC (*P* < 0.01).

### Upregulated expression of *Il1b* in the ileum and colon of rats with DIO decreased after CR and BS

**Fig. 2:**
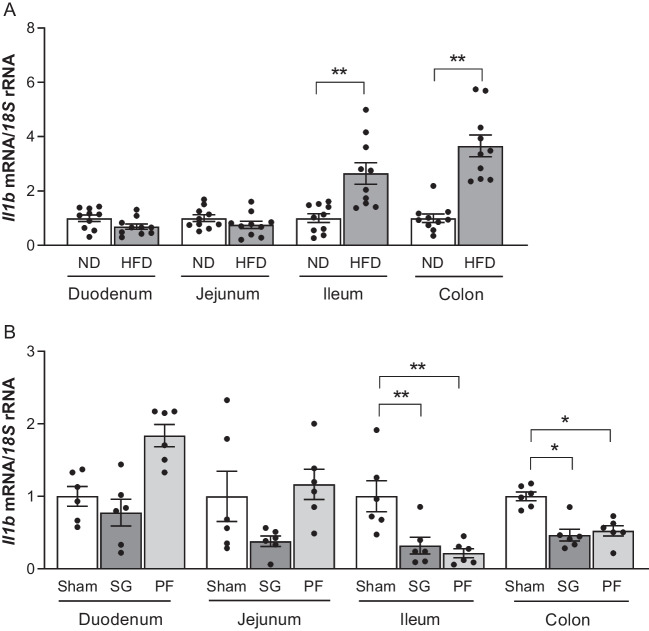
Gene expression levels of *Il1b* in the duodenum, jejunum, ileum and colon of rats under a (**A**) normal diet (ND, *n* = 10) and high fat diet (HFD, *n* = 10) as well as (**B**) after bariatric surgery [Sham, *n* = 6; sleeve gastrectomy (SG), *n* = 6; pair-fed (PF), *n* = 6]. All bar graph values are expressed as the mean ± SEM. Differences between groups were analyzed by an unpaired Student *t*-test, and a one-way ANOVA followed by Tukey’s *post hoc* tests. ^*^P < 0.05 and ^**^P < 0.01. SG, sleeve gastrectomy; PF, pair-fed

Since the expression of *IL1B* was increased in the colon from patients with CC, we aimed to further study the effect of BS and CR using a rat model with DIO. As expected, HFD feeding increased adiposity (ND-fed rats 558 ± 24 g *vs* HFD-fed rats 630 ± 15 g, *P* < 0.05) and induced insulin resistance as evidenced by higher insulin levels (ND-fed rats 5.4 ± 0.5 ng/mL *vs* HFD-fed rats 5.8 ± 1.1 ng/mL, *P* < 0.05) and HOMA index (ND-fed rats 1.3 ± 0.1 *vs* HFD-fed rats 1.5 ± 0.3 *P* < 0.05). After surgery, rats with DIO showed an improvement in body weight (*P* < 0.05), whole-body adiposity (*P* < 0.01) and insulin sensitivity (*P* < 0.05). In line with our previous results in human samples, gene expression levels of *Il1b* were upregulated in the colon of rats with DIO compared to those fed a ND (*P* < 0.01) (Fig. 2A). In this regard we also found increased levels (*P* < 0.01) of *Il1b* in the ileum of rats under a HFD (Fig. 2A). Importantly, *Il1b* mRNA levels in the colon (*P* < 0.05) and ileum (*P* < 0.01) decreased after weight and fat loss achieved by both SG and CR (*P* < 0.05). (Fig. 2B). No differences in the duodenum or jejunum were found neither due to HFD nor weight loss.

### ACM upregulated the expression of *IL1B* in HT-29 cells

**Fig. 3:**
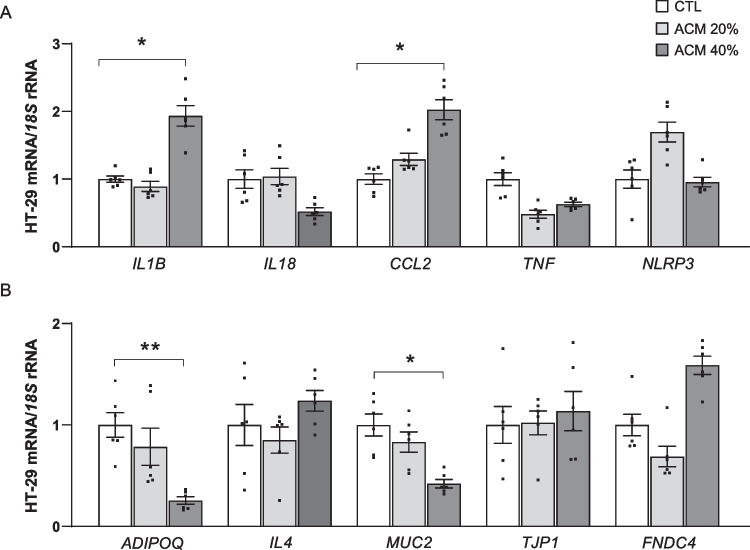
Effect of adipocyte-conditioned media (ACM) on the gene expression levels of *IL1B* as well as on inflammation- and intestinal integrity-associated markers. Bar graphs show the effect of ACM (20 and 40%) from individuals with obesity incubated for 24 h on the transcript levels of (**A**) interleukin *(IL)-1B*, *IL18*, monocyte chemoattractant protein-1 (*CCL2*), tumor necrosis factor-α (*TNF*) and nucleotide-binding oligomerization domain, leucine rich repeat and pyrin 3 (*NLRP3*) and (**B**) adiponectin *(ADIPOQ)*, *IL4*, mucin-2 (*MUC2*), tight junction protein 1 (*TJP1*) and fibronectin type III domain containing protein 4 (*FNDC4*). Values are the mean ± SEM (*n* = 5–6 per group). Differences between groups were analyzed by one-way ANOVA followed by Dunnett’s *post hoc* tests. ^*^P < 0.05 and ^**^P < 0.01

Since increased gene expression levels of *IL1B* were found in the VAT and in the colon from patients with OB and CC, we further explored if the adipocyte secretome from patients with OB affects the expression of *IL1B* as well as other factors related to inflammation and intestinal barrier integrity in HT-29 cells (Fig. 3). Gene expression levels of *IL1B* and monocyte chemoattractant protein-1 (*CCL2*) (both *P* < 0.05) increased after the treatment with ACM (Fig. 3A). Unexpectedly, a tendency towards a decreased mRNA levels of the pro-inflammatory gene tumor necrosis factor-α (*TNF*) and *IL18*, the main mediator of the NLRP6 inflammasome, was found after the treatment with ACM (Fig. 3A). Nonetheless, after ACM stimulation, mRNA levels of the anti-inflammatory adiponectin (*ADIPOQ*) were significantly downregulated (*P* < 0.01) together with levels of mucin-2 (*MUC2*) (*P* < 0.05), a key gene in the integrity of the intestinal barrier (Fig. 3B). No significant differences were found in the expression of tight junction protein-1 (*TJP1*) (Fig. 3B).

### IL-1β upregulated the expression of pro-inflammatory genes while it decreased the expression of anti-inflammatory factors in HT-29 cells

**Fig. 4:**
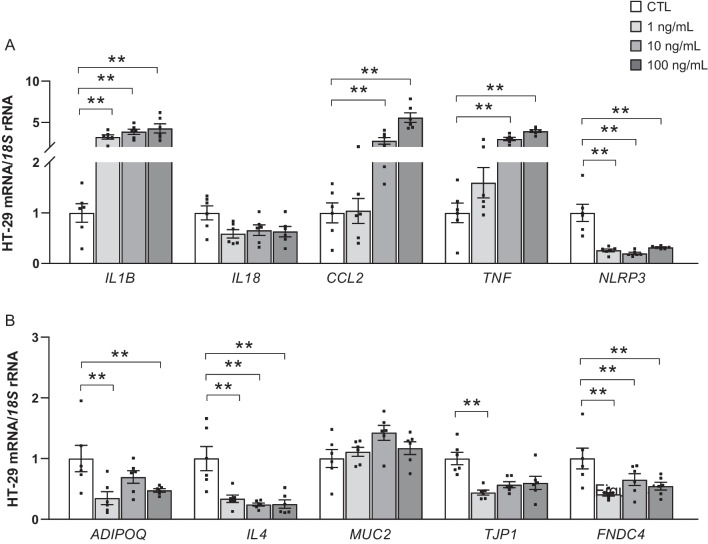
Effect of IL-1β treatment on the expression levels of (**A**) pro- and (**B**) anti-inflammatory markers in HT-29 cells. Values are the mean ± SEM (*n* = 5–6 per group). Differences between groups were analyzed by one-way ANOVA followed by Dunnett’s post hoc tests. ^**^P < 0.01. *ADIPOQ*, adiponectin; *CCL2*, monocyte chemoattractant protein-1; *FNDC4*, fibronectin type III domain containing protein 4; *IL*, interleukin; *MUC*, mucin-2; NLRP3, nucleotide-binding oligomerization domain, leucine rich repeat and pyrin; TJP1, tight junction protein 1; TNF, tumor necrosis factor-α

Since *IL1B* expression levels were upregulated in obesity, and the ACM also increased its expression levels in the colon adenocarcinoma HT-29 cell line, we aimed to further study its implication in obesity-associated CC. For that reason, we treated HT-29 cells with different concentrations of IL-1β (Fig. 4). The treatment with IL-1β strongly induced its own expression levels (*P* < 0.01) (Fig. 4A). We also found an upregulation of *CCL2* (*P* < 0.01), suggesting macrophage infiltration and accentuated tumor endothelial-mesenchymal transition as well as increased mRNA expression of *TNF* (*P* < 0.01) (Fig. 4A). Unexpectedly, mRNA levels of nucleotide-binding oligomerization domain, leucine rich repeat and pyrin 3 (*NLRP3*), fundamental in the processing of IL-1β, were downregulated after stimulation with IL-1β (*P* < 0.01) (Fig. 4A). This pro-inflammatory setting is further exacerbated by the decreased expression levels of the anti-inflammatory genes *IL4, ADIPOQ* and fibronectin type III domain containing protein 4 (*FNDC4*) after IL-1β treatment (*P* < 0.01 for all) (Fig. 4B). No significant differences were detected in the expression of *MUC2* but the mRNA of *TJP1* was downregulated after IL-1β treatment as shown in Fig. 4B.

## Discussion

**Fig. 5:**
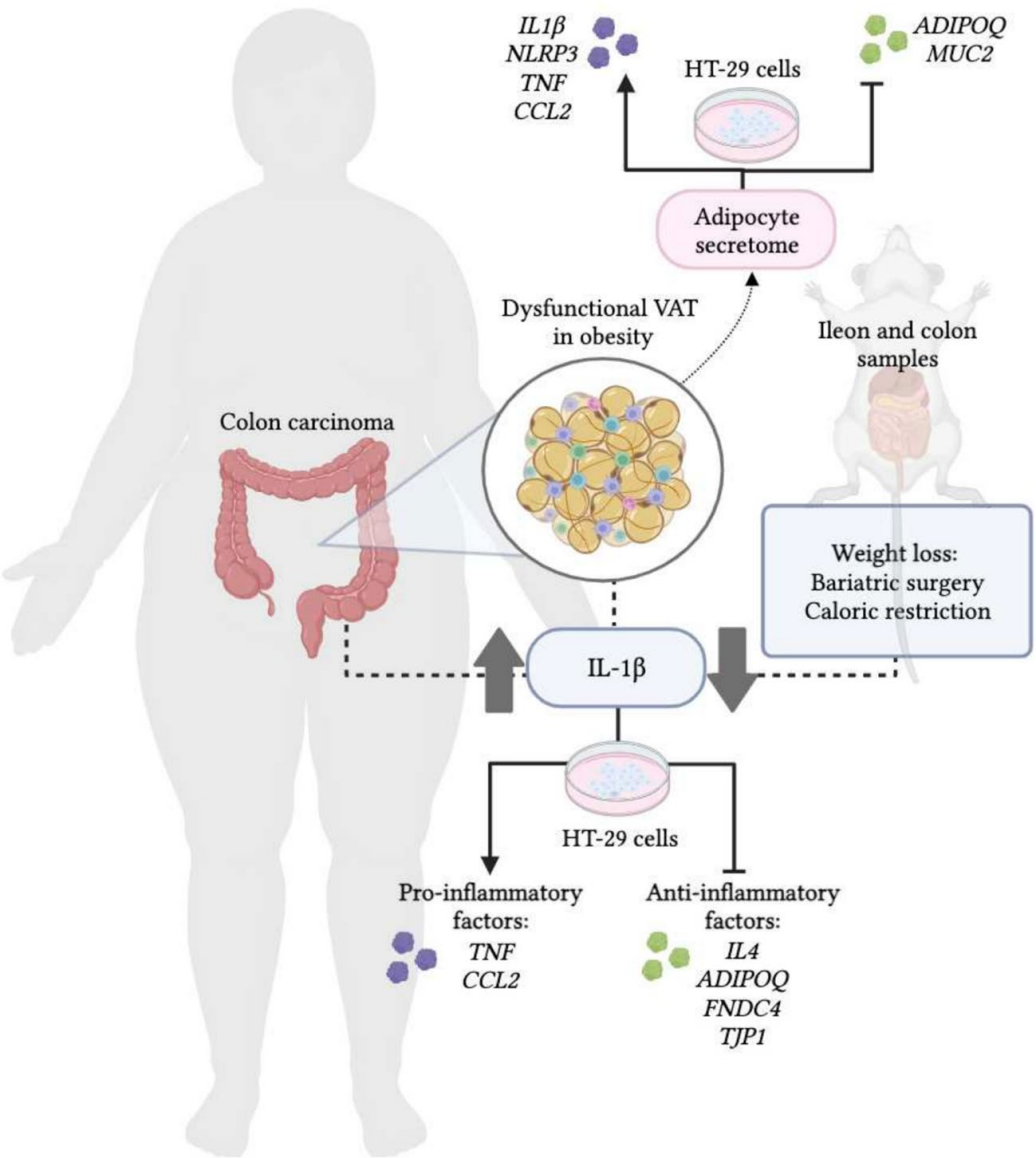
IL-1β is upregulated in VAT from patients with OB and CC and in the colon tissue from patients with CC. Weight loss achieved by either sleeve gastrectomy or caloric restriction downregulated the expression of *Il1b* in an animal model. In turn, IL-1β contributes to the inflammatory milieu in obesity-associated CC by upregulating pro-inflammatory factors such as *TNF* and *CCL2* and downregulating the anti-inflammatory factors as *IL4*, *ADIPOQ*, *FNDC4* and *TJP1*, mainly involved in the regulation of the intestinal barrier integrity. The treatment of the adenocarcinoma HT-29 cell line with the ACM obtained from patients with obesity upregulated the expression of pro-inflammatory *IL1B*, *NLRP3*, *TNF* and *CCL2* while downregulating anti-inflammatory *ADIPOQ* and *MUC2.* ACM, adipocyte conditioned media; *ADIPOQ*, adiponectin; *CCL2*, monocyte chemoattractant protein-1; *FNDC4*, fibronectin type III domain containing protein 4; *IL*, interleukin; *MUC*, mucin-2; *NLRP3*, nucleotide-binding oligomerization domain, leucine rich repeat and pyrin; *TJP1*, tight junction protein 1; *TNF*, tumor necrosis factor-α

Chronic inflammation is a hallmark in the development of obesity-associated cancers including CC. Different cytokines have been identified as having pro-inflammatory functions with high relevance in the contribution of the systemic low grade inflammation present in an obesity scenario [8, 35, 65]. Among the different cytokines involved in this process, we focused primarily on the effects of IL-1β in the development of CC. Our findings indicate that i) OB and CC upregulated gene expression levels of *IL1B* in VAT, ii) mRNA levels of *IL1B* were increased in the colon from patients with CC, iii) an upregulated expression of *Il1b* in the ileum and colon of rats with DIO decreased after CR and BS, iv) the secretome of visceral adipocytes from patients with OB increased the expression of *IL1B* in HT-29 cells together with a decrease of *ADIPOQ* and *MUC2*, and v) IL-1β modulated inflammation in HT-29 cells (Fig. 5).

VAT is a key tissue to study obesity-associated inflammation, since it is characterized by a complex inflammatory phenotype [65]. Epidemiological studies have established a relationship between intra-abdominal fat and metabolic alterations, which accentuates the risk of developing different chronic diseases including cancer [11, 49]. The upregulated levels of *IL1B* in the VAT from patients with OB and CC found in our study, are in accordance with previous results showing that obesity increases the release of IL-1β in AT [64]. In addition, a meta-analysis reported higher transcript levels of *IL1B* in AT samples obtained from patients with obesity compared with normal weight controls [1]. Moreover, we also observed an upregulation in the gene expression levels of *IL1B* in the colon from patients with CC. In this line, previous studies have shown an association between levels of IL-1β in the colon with intestinal inflammation and CC invasiveness [26]. Interestingly, upregulated tumorigenic expression of *Il1B* has been associated with a worse prognosis in patients with cancer [12]. A potential explanation for the relevance of IL-1β both at the AT and colon samples may be that increased levels of IL-1β have been proposed to contribute to the impairment of adipocytes by activating nuclear factor-κB (NF-κB) signaling, which in turn is key in the development of intestinal inflammation [33].

Based on the results obtained with human samples, we studied the expression levels of *Il1b* using a DIO rat model. The increased expression levels of *Il1b* in the ileum and colon of rats under a HFD are in line with the results obtained with the human samples, suggesting a pro-inflammatory environment in the colon from rats with obesity that may favor CC development. The effect of weight loss and BS on cancer mortality remains controversial. Some studies report a decrease in cancer incidence and cancer-related mortality after weight loss associated with BS [2, 51], while others have failed to obtain consistent evidence that demonstrates that weight loss interventions have a direct effect on cancer mortality [42]. Therefore, we aimed to further study the changes in the gene expression levels of *Il1b* after weight loss interventions such as BS or CR in a DIO rat model. Despite different types of BS being currently used, in this study we focused on SG due its technical simplicity, low complication rate and effective weight loss results [17, 51]. Gene expression levels of *Il1b* were downregulated after SG and CR interventions in ileum and colon samples. These findings are in line with previous results in mice with induced colitis, in which transcript levels of *Il1b* in colon tissues decreased in parallel to a decrease in body weight [30]. Additionally, further studies also reported reduced gene and protein expression levels of *Il1b* in the AT of DIO rats after BS [46] as well as decreased expression levels of *NLRP3* after weight loss achieved by either BS or CR in DIO rats [25]. Furthermore, serum levels of IL-1β have also been shown to decrease 6 months after BS [71].

To further study how obesity affects the expression of *IL1B* in a CC context, we treated the colon adenocarcinoma HT-29 cell line with increasing concentrations of ACM obtained from patients with OB. We found an upregulation in the transcript levels of *IL1B,* suggesting that the secretome of dysregulated adipocytes accentuates the inflammatory burden in a CC environment. Furthermore, the gene expression levels of *CCL2* were upregulated after the stimulation with ACM. CCL2 is a chemokine that primarily induces immune cell infiltration [32], contributes to inflammation and promotes angiogenesis [9]. The axis CCL2 and its receptor CCR2 has been established as an early marker of diagnosis and prognosis in different types of cancers [41]. Specifically, higher circulating levels of CCL2 have been found in patients with CC [48]. Further accentuating this inflammatory milieu, ACM downregulated the expression levels of *IL18,* a key factor for epithelial restoration [10, 52] and *ADIPOQ*. Adiponectin has been described as an anti-inflammatory adipokine, which promotes insulin sensitivity and protects against hepatic lipid accumulation [61]. In fact, adiponectin has been previously shown to be downregulated in patients with obesity [20] and a low adiponectin to leptin (Adpn/Lep) ratio suggests an increased cardiometabolic risk, systemic inflammation and oxidative stress [19]. We also found that obesity affects the intestinal barrier integrity by downregulating the expression levels of *MUC2*, an important factor that promotes barrier integrity by producing mucus and by protecting the intestine against pathogens [55]. In this line, *MUC2* alterations have also been linked to inflammation and carcinogenesis [70] with downregulated levels in the colon of patients with CC [25].

The direct effect of IL-1β on CC was also studied by analyzing its impact on the expression of factors related to inflammation and intestinal barrier integrity. We found that IL-1β treatment further upregulated its own expression levels. Interestingly, in parallel with this finding, an inverse relation between the concentrations of IL-1β and the expression of *NLRP3* was found. NLRP3 inflammasome is fundamental in the processing of IL-1β and previous studies have demonstrated its complex role in the development of CC [25, 54]. Depending on the context and the tissue where its location is analyzed, inflammasomes can either be considered pro- or anti-tumoral [25, 72]. In this line, while an overexpression of *NLRP3* has been shown in the VAT of patients with OB and CC being linked to increase inflammation [25], other studies have shown that blunting NLRP3 expression in animal models increases their susceptibility to develop CC [34]. Therefore, it suggests that the fine-tuning levels of NLRP3 is necessary for its optimal function, since a variation in its expression and function may either favor a pro- or an anti-inflammatory burden [34]. In line with the inflammatory scenario linked to CC, an enhanced expression level of *TNF*, a characteristic pro-inflammatory mediator, was detected after IL-1β treatment. TNF-α is a key factor in triggering an inflammatory immune response [69]. In fact, therapies targeting TNF-α have been highly efficacious against intestinal chronic inflammatory diseases such as ulcerative colitis and Crohn’s disease [16, 69]. We also found a direct relation between the concentration of IL-1β and the expression of *CCL2*. This sustained upregulation in the expression of *CCL2* both under treatment with ACM and IL-1β, highlights an important role of the aforementioned CCL2-CCR2 axis in obesity-associated CC carcinogenesis. Some studies have associated this axis with the expression of nonmalignant cells in the tumor microenvironment (TME) [32], while others have described that the inhibition of *CCL2* reduced tumor metastasis by affecting macrophage infiltration [63]. Currently in cancer research, the concept of TME has gained notable attention. TME refers to the interaction between the tumor and different cellular components that conform a metabolic niche which directly influences the tumorigenesis process [56]. Immune cells, including fibroblasts, mesenchymal stem cells, macrophages and others, are part of the TME and play a crucial role in carcinogenesis [63]. After tissue injury, as the one observed in both dysfunctional AT and colon cancer, monocytes are recruited and differentiate into macrophages at the specific tissue [3]. In a carcinogenic scenario, tumor-associated macrophages (TAMs) infiltrate the tumor and are influenced by cancerous cells to promote tumor growth [5, 50]. Depending on their polarization, pro-inflammatory M1 or anti-inflammatory M2, TAMs could either be pro- or anti-tumoral [50]. In this line, obesity reportedly induces the differentiation of pro-inflammatory macrophages, which in turn increases the production of IL-1β [73, 74]. Therefore, further studies should focus on studying how obesity-associated CC as well as IL-1β and CCL2 affect the macrophage differentiation directly in the TME. Regarding anti-inflammatory factors, IL-1β downregulated the expression of both *ADIPOQ* and *IL4*, further enhancing the inflammatory milieu in the carcinogenic environment. Additionally, a downregulation of *TJP1* and *FNDC4* was also observed. In this line, zonulin-1, the protein encoded by the *TJP1* gene, is essential for intestinal mucosal repair and is downregulated both at a transcript and protein level in intestinal samples from patients with inflammatory bowel disease [36]. In line with our results, FNDC4 has been previously described as an anti-inflammatory factor increased in inflamed sites of the intestine of patients with inflammatory bowel disease [4] as well as an adipokine with insulin-sensitizing [27] and anti-lipogenic properties [22]. In fact, FNDC4 protects adipocytes against inflammatory cell death caused by SARS-CoV-2 infection by blunting the activation of the NLRP3 inflammasome [21].

As limitations of this study, further cell lines should be used under the same conditions to further amplify our findings. The determination of the circulating levels of IL-1β in the animal model would contribute to better elucidate the role of weight loss in the regulation of this protein in the context of obesity and CC development. Additionally, the measurement of protein expression levels may shed information about full protein functionality.

Taken together, here we show that IL-1β contributes to the inflammatory milieu in obesity-associated CC, demonstrated by its upregulation in both the VAT from patients with OB and CC and in the colon tissue from patients with CC. Furthermore, IL-1β modulated inflammation in CC, by upregulating pro-inflammatory and downregulating anti-inflammatory factors (Fig. 5). Further studies to understand how IL-1β modulates the carcinogenic process in obesity-associated CC and to establish a relation between IL-1β and cancer prognosis are warranted.

## Supplementary Information

Below is the link to the electronic supplementary material.

Supplementary file1 (DOCX 17 KB)

## Data Availability

No datasets were generated or analysed during the current study.
