# Supplementary material for: Increased expression of IL-1β in adipose tissue in obesity influences the development of colon cancer by promoting inflammation
Source: J Physiol Biochem. 2024 Sep 21;81(2):471–82. doi: 10.1007/s13105-024-01048-5 (PMC12279602; doi:10.1007/s13105-024-01048-5)
Supplement: Supplementary file 1 — Supplementary file1 (DOCX 17 KB) [file 13105_2024_1048_MOESM1_ESM.docx]

**Supplemental Table 1.** Clinic and pathological characteristics of patients with colon cancer.

| **Gender, n**  Male  Female | 14  12 |
| --- | --- |
| **Location of primary lesion, n**  Transverse colon  Right hemicolon  Left hemicolon  Missing | 3  10  13  0 |
| **TNM stage, n**  I  II  III  IV  Missing | 4  2  12  4  1 |
| **Differentiation, n**  Well  Moderately  Poorly and undifferentiated  Missing | 2  17  3  1 |
| **Tumor size, n**  < 5 cm  > 5 cm  Missing | 13  6  4 |
| **Lymph node status, n**  Positive  Negative | 10  16 |

**Supplemental Table 2.** Sequences of the primers and TaqMan^®^ probes.

| **Gene (GenBank accession)** | **Oligonucleotide sequence (5’-3’)** | |
| --- | --- | --- |
| *ADIPOQ* (NM_001177800) |  | |
| Forward | GGAGATCCAGGTCTTATTGGTCCTA | |
| Reverse | CCTTGGATTCCCGGAAAGC | |
| TaqMan^®^ Probe | FAM-ACATCGGTGAAACCGGAGTACCCGG-TAMRA | |
| *CCL2* (NM_002982) |  | |
| Forward | GCTCATAGCAGCCACCTTCATT | |
| Reverse | TCTGCACTGAGATCTTCCTATTGGT | |
| TaqMan^®^ Probe | FAM-TCGCTCAGCCAGATGCAATCAATGC-TAMRA | |
| *FNDC4* (NM_022823.2) |  | |
| Forward | TGGTCATCATTGTGGTGGTGT | |
| Reverse | TGGCCTTCCCTGAGGACTCT | |
| TaqMan^®^ Probe | FAM-CATGTGGGCTGCTGTAATTGGGCTGTT-TAMRA | |
| *IL1B* (NM_000576) |  | |
| Forward | CAGTGGCAATGAGGATGACTTG | |
| Reverse | GTAGTGGTGGTCGGAGATTCGTA | |
| TaqMan^®^ Probe | FAM-TGGCCCTAAACAGATGAAGTGCTCCTTCC-TAMRA | |
| *IL4* (NM_001562) |  | |
| Forward | CCAAGGAAATCGGCCTCTATT | |
| Reverse | CCTCTAGGCTGGCTATCTTTATACATACT | |
| TaqMan^®^ Probe | FAM-TTCTGACTGTAGAGATAATGCACCCCGGAC-TAMRA | |
| *IL18* (NM_001562) |  | |
| Forward | CCAAGGAAATCGGCCTCTATT | |
| Reverse | CCTCTAGGCTGGCTATCTTTATACATACT | |
| TaqMan^®^ Probe | FAM-TTCTGACTGTAGAGATAATGCACCCCGGAC-TAMRA | |
| *MUC2* (NM_002457) |  | |
| Forward | ACGGCCTGCAGAGCTATTCA | |
| Reverse | TGATCTTCTGCATGTTCCCAAAC | |
| TaqMan^®^ Probe | FAM-ATTCCTCTCTGACGGCGTGCTCTTCAGT-TAMRA | |
| *NLRP3* (NM_001079821.2) |  | |
| Forward | AAGCTTCAGGTGTTGGAATTAGACA | |
| Reverse | GTTGCCCAGGCTCAGCTTT | |
| TaqMan^®^ Probe | FAM-CACACTGCTGCTGGGATCTTTCCACA-TAMRA | |
| *TJP1* (NM_003257.5) |  | |
| Forward | GCACAGCAATGGAGGAAACAG | |
| Reverse | TCTCGTCCACCAGATATTGCAAT | |
| TaqMan^®^ Probe | FAM-ACAACATACAGTGACGCTTCACAGGGCTC-TAMRA | |
| *TNF* (NM_000594) |  |  |
| Forward | CCCCAGGGACCTCTCTCTAATC |  |
| Reverse | ACATGGGCTACAGGCTTGTCA |  |
| TaqMan^®^ Probe | FAM-CCTCTGGCCCAGGCAGTCAGATCAT-TAMRA |  |
| *Rat Il1b* (NM_031512) |  | |
| Forward | ACAGAACATAAGCCAACAAGTGGTATT | |
| Reverse | GTGGGTGTGCCGTCTTTCAT | |
| TaqMan^®^ Probe | FAM-CAAGGAGAGACAAGCAACGACAAAATCCC-TAMRA | |

*ADIPOQ,* adiponectin; *CCL2*, monocyte chemoattractant protein-1; *FNDC4*, fibronectin type III domain containing 4; *IL*, interleukin; *MUC2*, mucin 2; *NLRP3*, nucleotide-binding oligomerization domain, leucine rich repeat and pyrin 3; *TJP1*, tight junction protein 1; *TNF*, tumor necrosis factor-α.
